# We Are Ageing

**DOI:** 10.1155/2014/808307

**Published:** 2014-06-22

**Authors:** Genovefa D. Kolovou, Vana Kolovou, Sophie Mavrogeni

**Affiliations:** Cardiology Department, Onassis Cardiac Surgery Center, 356 Sygrou Avenue, 17674 Athens, Greece

## Abstract

Ageing and longevity is unquestioningly complex. Several thoughts and mechanisms of ageing such as pathways involved in oxidative stress, lipid and glucose metabolism, inflammation, DNA damage and repair, growth hormone axis and insulin-like growth factor (GH/IGF), and environmental exposure have been proposed. Also, some theories of ageing were introduced. To date, the most promising leads for longevity are caloric restriction, particularly target of rapamycin (TOR), sirtuins, hexarelin and hormetic responses. This review is an attempt to analyze the mechanisms and theories of ageing and achieving longevity.

## 1. Introduction

Ageing and longevity is unquestioningly complex. The heterogeneity of ageing phenotype on the one hand and length of life span on the other, in subjects of the same species, are due to input of both genetic and environmental factors. While everybody is familiar with ageing, the ageing defining it is not so straightforward. Frequently, it has been defined as the collection of changes that reduce human length of life span.

The great interest in the ageing research which is observed lately has been inspired by lengthening of the average human life span (age at which 50% of a given population survives), lengthening of the maximum human life span (longest-lived member of the population), and elevated percentage of the elderly and very elderly in the populations [1]. Several thoughts and mechanisms of ageing such as pathways involved in oxidative stress, lipid and glucose metabolism, inflammation, DNA damage and repair, growth hormone axis and insulin-like growth factor (GH/IGF) [2], and environmental exposure [3] have been proposed. Also, some theories of ageing have been introduced.

Generally speaking, ageing humans die from age-related diseases, such as heart failure, myocardial infarction, stroke, diabetes mellitus, cancer, Alzheimer's, Parkinson diseases, osteoporosis, and osteoarthritis. Lately, many tactics have been proposed for enhancing human longevity [4] with varying degrees of success. Such example is a caloric restriction [5, 6], hormonal replacement [7], and antioxidant treatment [8, 9]. Also, some strategies for enhancing human longevity were introduced, for example, engineered negligible senescence proposed by De Grey [10] and nucleic acid therapy; see review by Lai [11]. Last 2 decades, the genes responsible for ageing or genes that promote longevity have also begun to be cloned [12, 13].

In this review, the epidemiological and clinical studies according to ageing, longevity, and exceptional longevity as well as the ageing theories, gender differences, and successful and unsuccessful ageing will be analyzed.

## 2. Epidemiological Studies of Ageing

Two centuries ago, the human average life span was below 40 years. Fortunately, nowadays, the life span rose to approximately 80 years in many developed countries (see details at http://www.mortality.org) [14, 15].

## 3. Causes of Ageing (Primary and Secondary)

Causes of ageing can be simplified as primary and secondary. Primary ageing is not attributed to a specific cause and is referred to currently expected sum of changes, physiological, genetic, and molecular, that occurs with the passage of time from fertilization to death (slow movements, declining vision and hearing, inability to adapt to stress, decrease in resistance to infections, and others). The aggravated causes of ageing can be called as secondary causes. For example, secondary ageing processes result from degenerative diseases (mentioned above) and poor health practices (lack of exercise, smoking, excess fat ingestion, and other forms of self-damage).

### 3.1. We Are Ageing

We cannot escape the physical nature of life and the direct relationship of genetics, enviromental influences (mass/energy and gravity plays a significant role in all life processes), chemistry, and generally the fundamental physics of the universe. There are several theories of ageing which have been developed in the last century.

#### 3.1.1. Theory of Evolutionary Ageing

When words such as life or ageing are so obvious and at the same time so indefinable that no definition is provided in the scientific literature, it is frequently useful to look into the words we routinely use when talking about them. Thus, life can be defined as accidental changes in the store of information, which raise the chance of surviving. Furthermore, life means the ability to adapt to one's environment and its own limitations. On the other hand, we have accepted that ageing is the unchangeable component of our life.

There are 4 main concepts in current evolutionary gerontology based on classical evolutionary process concepts. In 1889, August Weismann theorized that ageing is part of life's program because the old need to remove themselves from the place to make space for the following generations. In 1952, Peter Medawar suggested (*Medawar's theory*) that ageing is a subject of neglect (late-acting deleterious mutations can remain in the population simply because natural selection does not act against them) [16]. He believed that nature is a very competitive place, and practically all species die before they reach old age. Thus, there is not any reason why their bodies should stay fit for long time. Particularly, almost all will be killed by predators or disease or by accident. In 1957, Williams further developed Medawar's theory. He proposed the theory called* antagonistic pleiotropy* (one gene that has two or more effects on the phenotype) [17]. Generally, this means that genes, which were favorable early in life, will cost us later on. He also suggested that senescence might cause many deaths. For example, in the earliest stages of senescence, even a small loss of speed movement can cost the animal to be caught by predators, while younger animals can run away successfully. In 1977, Kirkwood proposed a third theory of ageing called* disposable soma theory* (body must budget the amount of energy available to it) [18]. Natural selection encourages the spread of traits that enable a higher energy investment in reproduction, which comes at the expense of a lower energy investment in maintenance processes. This is not always a case. For example, the energy required for body preservation and repair is relatively minor compared to the energy required for gestation. Yet, the male animals have similar or even decreased life spans compared to females who need higher energy (gestation and other reproductive activities). The fourth theory is* the negative senescence theory* introduced by Lee in 2003 [19] and by Vaupel et al. in 2004 [20]. They believed that natural selection forces could be effective in postreproductive individuals when intergenerational transfers take place.

As a final point, the stochastic factors that influence the lifespan should be mentioned. Even genetically identical subjects, who are grown in a common environment, do not have the same life span [21].

#### 3.1.2. Cellular Theory of Ageing

Hayflick in 1974 introduced the* Hayflick limit*, suggesting that primary mammalian cells grown in culture have a finite replicative capacity and when the cells achieve their greatest number of divisions, they undertake growth arrest and become senescent [22]. Another molecular theory is the* telomere shortening theory*; see review by Tzanetakou et al. [23], which suggests loss in telomere length with every cell division.

*Replicative senescence or cellular senescence* is weakening and dyeing of cell after several numbers of times that cells have been divided [23]. It is noteworthy to mention the apoptosis hypothesis.* Apoptosis* is a programmed cell death, which is responsible for killing infected, cancer, and other abnormal cells. Thus, the presence of apoptosis should not be problematic for ageing. However, later in life, the apoptosis can affect also the healthy cells. This hypothesis can be viewed as a special case of the antagonistic pleiotropic theory; see above. Oxidative stress is also believed to be a major mediator, since cellular senescence can be prematurely triggered by various traumatic stimuli such as DNA injury and abnormal oncogene activity [24].

### 3.2. Gender, Ethnicity, and Ageing/Longevity

#### 3.2.1. Gender

In most countries, women live longer than men [25]. Worldwide, 75% and 90% of very elderly individuals (aged 100 years and 110 years, resp.) are women. This has occurred due to the following. (1) Lower mortality: the mortality rate is lower in childhood and adolescence women compared with men at the same age [25]. The cause of death in young men is frequently due to accident (motorcycling, fighting, diving, and other unsafe activities). (2) Evolutionary theory: a high accidental death rate determines fast ageing (men do not live long enough to experience ageing) [26, 27]. (3) Mammalian target of rapamycin (mTOR): testosterone activates the mTOR pathway [28] and in turn the mTOR stimulate cellular growth and hypertrophy [29]. Thus, the activation of mTOR may offer a benefit in early life for which men have to pay by accelerating ageing later. (4) Presence of two copies of X chromosomes: the occurrence of 2 X chromosomes in women allows additional cell viability and proliferative capacity.

#### 3.2.2. Ethnicity

The ethnic differences in ageing and longevity are still not very well documented. It is possible that some of the differences in ageing among ethnic groups may be explained by discrepancies in healthcare, environmental and economic status, and life occupation [30].

Also, genetic factors that play major role in ageing and longevity are different among subjects with different ethnicity. Many metabolic-regulating genes were found to correlate with longevity according to the ethnicity [31]. Barzilai et al. [32] suggested that exceptional longevity was associated with approximately a 3-fold increased frequency of homozygosity V of* CETP* (cholesterol ester transport protein) I405V gene in the Ashkenazi Jewish. Soerensen et al. [33] found that* CETP* gene polymorphism is associated with longevity in oldest-old (ages 92-93) Danes population. Our research group [31] have not found any differences in genotypes/allele frequency of both (TaqB1 and I405V)* CETP* gene polymorphisms in Greeks. Similarly, Cellini et al. did not confirm the association between the* CETP* I405V variation and the healthy aging phenotype in Italians [34].

### 3.3. Genes Increasing Life Span or Reducing Ageing

Generally, it is accepted that longevity referred to someone who lives longer than the age of 90 years. The family longevity can be considered in the family irrespectively of whether the individuals still living were over the age of 90 years [35]. Some studies are using family longevity selection score (FLoSS), which is a summary measure based on the survival experience of the oldest living generation of siblings relative to what would be expected based on birth cohort life tables [36]. The exceptional longevity refers to survival outcomes and longevity that does not have any threshold; this can be with/without disease or disability. The disability and/or morbidity towards the end of very long lives have been found to be strongly familial [37]. Usually, the exceptional longevity referred to a person who lives in good health and much longer than 91 years of age for males and 95 for females. Survival to age of 100 is an uncommon occurrence (2 men and 14 women out of 1000 from 1900 birth cohort survived to age 100).

On the basis of demographic data, Avery et al. [38] proposed two terms of exceptional longevity: relative and absolute. Relative exceptional longevity suggests that longevity is concept country/population specific and must take into consideration the life expectancy of the different populations/countries, which show great variability owing to historical, anthropological, and socioeconomic differences. In the absolute exceptional longevity term, longevity could be defined according to the maximum life span attained and scientifically validated by human beings in the planet.

The genes, which are associated with longevity such as insulin-like growth factor 1 (*IGF1*), IGF1 receptor (*IGF1R*), phosphatidylinositol 3-kinase (*P13K*),* PTEN* (phosphatase and tensin homolog),* AKT*, and* FOXO* (forkhead box transcription), have primary roles in other physiological procedures and particularly in signal transduction [39]. Thus, it seems that natural selection does not affect genes that cause ageing, but ageing occurs as a consequence of pleiotropic effects of genes that are involved in other procedures [40]. Most life-extension effects have been found to result from* hypomorphic *(reduction in gene (protein, RNA) expression, but not a complete loss) or* nullomorphic* (complete loss of function) mutations, which means that the wild-type gene shortens life span [39]. Thus, the wild-type genes (gerontogenes) indicate a negative effect on life span longevity and blocking their expression should increase the life span. Oppositely, for longevity genes, the* nullomorphic *alleles result in life shortening.

Furthermore, the gerontogene mutants show decreased strength and fail to compete with wild-type animals [41]. On the other hand, the longevity mutations increase the ability to handle oxidative stress and starvation [42].

#### 3.3.1. Genes Associated with Longevity

In addition to testing, genes known to be associated with age-related diseases and phenotypes for association with longevity and genes known to promote longevity in model organisms have been examined in human populations. The insulin-signalling pathway negatively regulates the FOXO factor [43]. When insulin or IGF signalling is low, FOXO is activated and life span extension occurs [44]. An overrepresentation of rare* IGF1R* mutations has been observed in centenarians [45]. These mutations are associated with reduced activity of IGF1R as measured in transformed lymphocytes [45].

The* FOXO3A* alleles were associated with longevity in Asian and European populations [46]. It is noteworthy to mention that the alleles associated with longevity are unlinked to known coding single nucleotide polymorphisms (SNPs), so the functional SNPs may affect gene expression rather than protein activity. Willcox et al. [44] found that common, natural genetic variation within the* FOXO3A* gene was strongly associated with human longevity and was also associated with several phenotypes of healthy aging [44]. In two replication studies, the odds ratios of the* FOXO3A* alleles were 1.26 for a German population and 1.36 for a Chinese population [46, 47]. These alleles in long-lived individuals may promote better health and contribute towards extended life span by increasing expression or activity of FOXO3A.

Genes associated with human longevity that have been replicated in various populations are* FOXO3A *and* APOE* [46–48]. Flachsbart et al. [46] extended the initial finding observed in Japanese men to women and found that both genders were likely to be equally affected by variation in* FOXO3A* and suggested as a susceptibility gene for prolonged survival in humans. Also, replication in a French centenarian sample produced a trend that supported the previous results [46]. However, these genes account for only a small portion of the genetic contribution to longevity measured through family heritability studies [49]. Thus, much of the heritability of life span remains to be clarified.

## 4. Successful Ageing

The rate of ageing may not be synchronous with chronologic time. Elderly individuals have very heterogeneous health phenotypes. A significant number of the elderly are functionally independent and are surviving well into their 9th, 10th, and even 11th decade of life. Nowadays, there is no shortage of definitions concerning life and successful ageing. Particularly, there are evidences that interventions such as physical activity can reduce disability and promote better health in late life, and morbidity becomes compressed into a much shorter duration [50]. It seems that longer-lived species possess several mechanisms for offsetting damage (oxidation, telomere shortening, and others) more effective than those of shorter-lived species.

There are several factors associated with successful ageing. One very important factor is the personality, which has been linked to health outcomes and longevity [51]. Kato et al. [52] evaluated the personality of centenarians by the personality outlook profile scale and demonstrated that the centenarians may share particular personality characteristics. They suggested that genetically based aspects of personality might play an important role in achieving positive health outcomes and exceptional longevity. Christensen et al. [53] studied health and performance assessment of Danish elders who were born in 1905 and reported the functional independence (physical and cognitive performance) as definition of successful ageing. Fried et al. [54, 55] have developed and implemented standardized tests of physical and cognitive function measured in a large cohort of studies such as the cardiovascular health study [54, 55]. The results on the survivors of this study were published by Newman et al. [56]. They reported that measurement of physical and cognitive function, rather than a simple count of comorbid conditions, is a key component for a definition of successful ageing. Similar results were found by Long Life Family and the Framingham studies [57, 58], the Swedish Centenarian study [59], and the Honolulu Heart study [60].

Thus, the successful aging can be characterized by avoidance (or late onset) of diseases and disabilities and preservation of desirable cognitive and physical function and social activities all through the life span.

## 5. Unsuccessful Ageing

Unsuccessful ageing is characterized by frailties, cognitive decline, and diminishing of executive function [61]. Many conditions have been identified to be responsible for decline of old person. Population-based studies performed in healthy elderly demonstrated that only 30% of population examined could be defined as successfully aged [62]. Although disabilities such as heart disease, diabetes mellitus, hypertension, and infections are regularly assessed in ageing individuals with possible stabilization, the cognition and neuropsychiatric disorders in healthy elderly are infrequently evaluated [63]. Particularly, conditions of impaired cognition and of apathy on nondemented healthy elderly are underestimated, especially that these conditions can be responsible for reduced autonomy and increased carelessness and progressive worsening of comorbidities [64]. Also, obesity and/or arthritis are significantly related to decreased active life expectancy and higher degrees of disability [65]. Similarly, the osteoporotic fracture (frequently present in elder noneasy mobile individuals) results in early death or loss of mobility [66]. Additionally, the elderly with Alzheimer's disease demonstrate reduced life expectancy [67].

## 6. Adaptability

The unique functional flexibility of centenarians suggests programming of biological pathways that promote well-being in old age. The long-term follow-up of the very elderly populations has provided evidences for adaptive capacity of certain physiologic systems and promoted long-term survival beyond reproduction [68]. For example, although ageing is related to muscle fiber atrophy and declined number of motor units, there is compensatory increased size of the average motor unit, innervation, and number of acetylcholine receptors per motor unit [69]. Also, although the decline of brain volume is observed with ageing, Venkatraman et al. [70] have shown compensatory increased functional activity in the right posterior parietal cortex among elders with high cognitive function. Also, Kantarci et al. [71] reported that memory, language, attention, and executive functions among elders without dementia are correlated with increased levels of activity of the cingulum of posterior cortex.

## 7. Familial Components of Longevity

There are data which reported that longevity is heritable, with heritability ranging from 20%–30% [72] to approximately 50% [73]. Also, the factors such as smoking, body mass index, and mortality are very crucial for human life span [72].

However, aging is not always hereditary and the aging process may be different among generations in families. As is mentioned earlier, many factors can contribute to way of aging and to life expectancy. Human cells have processes such as DNA methylation, histone modifications, and ncRNA that can be regulated by epigenetic modifications [74]. Longevity depends, also, on the environment exposure during life span and through changes that may occur to the epigenome that affect the rate of aging [74].

## 8. Candidate Genes 

The possible candidate genes responsible for ageing and longevity in humans are usually identified previously in animal models [75, 76]. The biggest problem with this procedure is that some gene families containing one gene in an animal model have several human homologs [77]. Another approach for identifying candidate gene is to study the gene expression (changes with ageing), which also has same limitations (choice of a tissue, studded group) [78]. The range of genes involved in the ageing until now seems to be widespread [79].

The mutation in the* age-1 gene* in* Caenorhabditis elegans* (free-living and transparent roundworm) was the first to be identified as longevity mutant [80]. The* age-1 gene* encodes PI3K [81] which has a key role in a signaling pathway (homologous to the mammalian IGF1 pathway) and eventually targets the transcription factor FOXO. FOXO regulates the expression of several genes that mediate stress resistance, metabolic processes, and toxin degradation [82].

## 9. Ageing Studies

Genetic studies of human life span have several advantages and disadvantages. The study cohorts usually vary and involve cohorts such as individuals born 90–100 years ago, after adolescence, same-sex twins, deaths after 90 years old, families with individuals with extreme longevity, and others.

Furthermore, the studies have various study designs such as linkage analysis (genetic mapping in humans), candidate-gene association studies, and longitudinal studies. The difficulty with linkage analysis according to longevity studies is that it requires large cohort study. Concerning the candidate-gene association studies, they usually compare the genotypes of subjects with exceptional longevity with those of the younger, which also have same limitations with the most serious lack of replication. In the longitudinal studies, the study cohort is followed for years. This kind of studies is less prone to biases than candidate-gene association studies.

### 9.1. Centenarian Studies

One approach to find candidate gene of ageing is to search for genetic differences between centenarians and average-aged individuals. The alleles with higher frequency in centenarians possibly affect genes that are important for longevity. A genome-wide scan for such predisposing loci was conducted by using 308 individuals belonging to 137 sibships demonstrating exceptional longevity. By using nonparametric analysis, significant evidence for linkage was noted for chromosome 4 [83]. Geesaman et al. [84] from the same research group tested the same variant in a second cohort of long living individuals from France, and this association was not replicated. However, genotyping 2000 single nucleotide polymorphisms (SNPs) within this 12 Mb locus revealed an association between microsomal transfer protein (MTP) and human life span [84]. This finding also could not be replicated in French or German populations [85].

The majority of studies comparing the genotypes of long-lived individuals to those of average-aged individuals are rather candidate-gene approaches than genome-wide analysis. For example, the *ɛ*4 allele of apolipoprotein E (*APOE*) gene is well known to increase risk of cardiovascular disease and Alzheimer's disease [48, 86]. The *ɛ*4 allele was found in lower frequency in nonagenarians and centenarians suggesting that individuals with this allele do not live as long as those without it [87]. Oppositely, the frequency of *ɛ*2 allele is increased in long-lived individuals and may offer a protective effect for cardiovascular disease and Alzheimer's disease [86, 87].

Atzmon et al. [88] genotyped 213 Ashkenazi Jewish centenarians, their offspring, and an age-matched Ashkenazi control group for 66 polymorphisms in 36 candidate genes related to cardiovascular disease. They found that the prevalence of homozygosity for the −641C allele in the* APOC3* promoter (rs2542052) was higher in centenarians (25%) and their offspring (20%) than in controls (10%). This genotype was associated with significantly lower serum levels of* APOC3* and a favorable pattern of lipoprotein levels and sizes [88].

Our group has evaluated in nonagenarians, centenarians, and middle-aged individuals the angiotensin-converting enzyme (*ACE*) gene, which is an important gene of the renin-angiotensin-aldosterone system (RAAS) that has been involved in the pathogenesis of hypertension, coronary artery disease, heart failure, and recently longevity [31]. We found that the I alleles of* ACE* gene were more frequent in centenarians compared to nonagenarians and controls. However, Yang et al. [89] did not find any association between* ACE* gene polymorphism and longevity in a Han Chinese population. Similarly, Blanché et al. [90] in a French centenarian cohort did not confirm the* ACE* gene association with longevity and discussed the risk of reporting false positive associations.

One of the theories of ageing is telomere shortening (see above). Telomeres are noncoding double-stranded repetitive structures at the ends of mammalian chromosomes [23]. Among other functions, they prevent chromosome degradation and maintain genome integrity. Telomeres become shorter with each cell division and once reach a crucial length they become dysfunctional and are no longer protective towards chromosomes. Shortening of the telomeres at the ends of chromosomes has been associated with age-related disease and mortality [91]. A recent study identified a common haplotype of four SNPs in the human telomerase reverse transcriptase gene (*hTERT*) that is present more frequently in centenarians and is associated with longer telomere length [92]. It was also shown that centenarians and their offspring maintain longer telomeres compared with controls and that longer telomeres are associated with protection from age-related diseases, better cognitive function, and lipid profiles of healthy ageing [93].

Some of the most frequent studied genes concerning human longevity were summarized in Table 1.

## 10. Management

To date, the most promising leads for longevity are caloric restriction [5, 6], targeting of rapamycin (TOR) [123], sirtuins [124], hexarelin [125], and hormetic responses [126]. Treated mice with resveratrol histone deacetylase sirtuin-1 (HDAC SIRT1) and high-fat diet increase life span, apparently mimicking the well-established contribution of caloric restriction to longevity [127].Chromatin-modifying agents are currently being tested as novel cancer therapies [128].

### 10.1. Glycogen Synthase Kinase-3 (GSK-3)

The GSK-3 family of serine/threonine kinases was first purified and then later cloned by Woodgett [129]. Its action is to phosphorylate and negatively regulate glycogen synthase (the rate-limiting enzyme in glycogen synthesis) [130, 131]. The substrates that are phosphorylated by GSK-3s can be classified into four categories: metabolic enzymes, signaling molecules, structural proteins, and transcription factors, typically involved in regulating cell proliferation and differentiation, cellular metabolism, cell survival, and cell cycle regulation [132]. In addition, GSK-3 has been related to several chronic diseases such as diabetes and Alzheimer's disease. However, it was not clear whether GSK-3 might regulate aging [132].

Zhou and Force [132] through targeting GSK-3*α* in mice found that there was acceleration in development of age-related pathologies in multiple organ systems (bone/skeletal system, gut, and liver), followed by increased inflammatory cytokines.

In addition, Zhou et al. [133] found that GSK-3*α* is a critical regulator of mTORC1, autophagy, and aging. When GSK-3a is absent, aging/senescence is accelerated in multiple tissues [133]. A potential medical therapy to elderly people could maintain GSK-3*α* activity and/or inhibit mTOR, in order to delay the appearance of age-related pathologies.

### 10.2. Cholesteryl Ester Transport Protein (CETP) Inhibitors

The CETP may have pro- or antiatherogenic properties depending upon the lipid metabolic setting [31, 134]. High-density lipoprotein (HDL) particles are influenced by CETP activity; CETP promotes the exchange of cholesteryl esters for triglycerides (TGs) between HDL particles and TG-rich lipoproteins [31, 135]. Ordovas et al. [136] suggested that increased HDL cholesterol levels resulting from lower CETP activity seem to be associated with a lower risk of coronary heart disease in men. Furthermore, reducing CETP concentration avoids the formation of small dense low density lipoprotein particles (LDL) [137].

The most common therapeutic strategies for coronary artery disease include 3-hydroxy-3-methylglutaryl-CoA reductase inhibitors (statins). Statins are of significant benefit in the primary and secondary prevention of atherosclerosis [138]. Statins slow atherosclerosis progression and can even induce atherosclerosis regression [139]. Moreover, statins can reduce the level of CETP; see review by Kolovou et al. [137].

CETP inhibition appears to be one particularly promising strategy, although they still have some difficulties; see review by Schaefer [140]. The CETP inhibitor torcetrapib increases plasma HDL cholesterol levels from 40% to 60%, while modestly decreasing LDL cholesterol [139]. Combining the HDL cholesterol-elevating properties of a CETP inhibitor with the LDL cholesterol-lowering properties of a statin may offer theoretically improved outcomes over targeting LDL cholesterol alone [139]. Toth [141], based on a number of considerations, including the complex relationship between loss of function mutations in* CETP* and risk for coronary artery disease and the clinical experience with torcetrapib, suggested that it is difficult to predict if CETP inhibition will be associated with reductions in rates of atherosclerosis disease progression and risk for cardiovascular events.

### 10.3. Glucagon-Like Peptide 1 (GLP-1)

Cells of enteroendocrine system are stimulated when food is entered and release GLP-1 in blood [142]. GLP-1 action is to bind and activate receptors on pancreatic *β*-cells, which produce and release insulin [143]. Muscle and liver cells are more sensitive to insulin through GLP-1, which has an antidiabetic effect [144]. Additionally, GLP-1 has the capability to cross blood brain barrier and stimulate cells in the hypothalamus causing suppression of appetite (anorexic effect) [142].

GLP-1 acts in many sites resulting in reduced circulating glucose levels and for that reason GLP-1 can be a potential therapeutic strategy for type 2 diabetes mellitus [142]. The problem is that GLP-1 has short half time in blood because it is cleaved and inactivated by dipeptidyl peptidase-4 (DPP-4) [145] making it impractical for routine use in patients. Developing peptide analogs of GLP-1 made the solution that are resistant to inactivation by DPP-4, such as exendin-4 (exenatide) is used successfully in glucose regulation in diabetic patients [146].

Moreover, GLP-1 acts through the GLP-1 receptor, which is present in large amounts in gastrointestinal system but has also been detected at lower levels in other tissues (nervous system, heart, vascular smooth muscle, and endothelial cells) [147–149]. When GLP-1 receptor is activated, it can trigger at least 2 intracellular pathways: (1) generation of the second messenger of cyclic adenosine monophosphate (cAMP) followed by activation of protein kinase A (PKA) and (2) indirect activation of epidermal growth factor receptor followed by phosphoinositide 3-kinase (PI3K) and Akt signaling [150].

Oeseburg et al. [151] found that GLP-1 could directly attenuate endothelial senescence. In addition, GLP-1 treatment had a protective effect on ROS-induced senescence in HUVEC cells and this was associated with a reduction in DNA damage, further supporting a protective effect of GLP-1 [151]. In pancreatic *β*-cells, GLP-1 reduced apoptosis, stimulated survival and proliferation, and increased insulin secretion [151]. The activation of downstream cAMP/PKA and PI3K/Akt signaling pathways in these cells primarily results in these effects of GLP-1 [152–154].

In conclusion, the longevity can be achieved by delay of apoptotic pathways. Various molecular factors with significant role in cell procedures may be potential molecular biomarkers and treatment targets that promote longevity. It is necessary to investigate and focus on the mechanisms that can preserve cells life. Numerous case-control candidate studies have evaluated the associations of the longevity with biologically plausible genes but results from these investigations are still tough to validate. These findings emphasize the importance of conducting large-scale studies with adequate replication.

## Figures and Tables

**Table 1:** Some of the most frequent studied genes concerning human longevity.

Gene	References	
*CETP *	Barzilai et al. 2003	[32]
	Atzmon et al. 2005	[94]
	Cellini et al. 2005	[34]
	Novelli et al. 2008	[95]
	Sanders et al. 2010	[96]
	Soerensen et al. 2013	[33]
	Kolovou et al. 2014	[31]
	Sun et al. 2013	[97]

*IGF* pathway	Bonafè M et al. 2003	[98]
	Suh et al. 2008	[45]
	Albani et al. 2011	[99]
	Soerensen et al. 2012	[100]

*FOXO* family	Bonafè et al. 2003	[98]
	Willcox et al. 2008	[44]
	Soerensen et al. 2010	[101]
	Zeng et al. 2010	[102]
	Kleindorp et al. 2011	[103]

*ACE *	Schachter et al. 1994	[87]
	Faure-Delanef et al. 1998	[104]
	Panza et al. 2003	[105]
	Forero et al. 2006	[106]
	Nacmias et al. 2007	[107]
	Blanchè et al. 2001	[90]
	Petranović et al. 2012	[108]
	Fiuza-Luces et al. 2011	[109]
	Kolovou et al. 2014	[31]

*SIRT1 *	Flachsbart et al. 2006	[110]
	Kuningas et al. 2007	[111]
	Kim et al. 2012	[112]
	Huang et al. 2013	[113]

*Klotho *	Arking et al. 2002	[114]
	Arking et al. 2005	[115]
	Invidia et al. 2010	[116]

*hTERT *	Atzmon et al. 2005	[94]
	Atzmon et al. 2010	[93]

*APOE *	Kervinen et al. 1994	[48]
	Schachter et al. 1994	[87]
	Blanché et al. 2001	[90]
	Wang et al. 2001	[117]
	Panza et al. 2003	[105]
	Feng et al. 2011	[118]
	Nebel et al. 2011	[119]
	Sebastiani et al. 2012	[120]
	Soerensen et al. 2013	[33]
	Schupf et al. 2013	[121]
	Beekman et al. 2013	[122]

IGF-1R: insulin-like growth factor 1 receptor, CETP: cholesteryl ester transfer protein, FOXO: forkhead box transcription, SIRT 1: sirtuin 1, hTERT: human telomerase reverse transcriptase, Apo: apolipoprotein, ACE: angiotensin-converting enzyme.
